# Improved two-stage model averaging for high-dimensional linear regression, with application to Riboflavin data analysis

**DOI:** 10.1186/s12859-021-04053-3

**Published:** 2021-03-25

**Authors:** Juming Pan

**Affiliations:** Department of Mathematics, Rowan University, Glassboro, NJ 08028 USA

**Keywords:** High-dimensional regression, Model averaging, Variable selection, Cross-validation, Jackknife

## Abstract

**Background:**

Model averaging has attracted increasing attention in recent years for the analysis of high-dimensional data. By weighting several competing statistical models suitably, model averaging attempts to achieve stable and improved prediction. In this paper, we develop a two-stage model averaging procedure to enhance accuracy and stability in prediction for high-dimensional linear regression. First we employ a high-dimensional variable selection method such as LASSO to screen redundant predictors and construct a class of candidate models, then we apply the jackknife cross-validation to optimize model weights for averaging.

**Results:**

In simulation studies, the proposed technique outperforms commonly used alternative methods under high-dimensional regression setting, in terms of minimizing the mean of the squared prediction error. We apply the proposed method to a riboflavin data, the result show that such method is quite efficient in forecasting the riboflavin production rate, when there are thousands of genes and only tens of subjects.

**Conclusions:**

Compared with a recent high-dimensional model averaging procedure (Ando and Li in J Am Stat Assoc 109:254–65, 2014), the proposed approach enjoys three appealing features thus has better predictive performance: (1) More suitable methods are applied for model constructing and weighting. (2) Computational flexibility is retained since each candidate model and its corresponding weight are determined in the low-dimensional setting and the quadratic programming is utilized in the cross-validation. (3) Model selection and averaging are combined in the procedure thus it makes full use of the strengths of both techniques. As a consequence, the proposed method can achieve stable and accurate predictions in high-dimensional linear models, and can greatly help practical researchers analyze genetic data in medical research.

**Supplementary Information:**

The online version contains supplementary material available at 10.1186/s12859-021-04053-3.

## Background

Model averaging and model selection are two important techniques for estimation and prediction in statistical modeling. Model selection is appealing for its simplicity and interpretability thus has been attracting more attention. A systematic review on model selection can be found in Heinze, Wallisch, and Dunkler [14] or Lee, Cho, and Yu [18]. The main drawback for model selection, however, is that the uncertainty is essentially ignored once an optimal model is found. A possible consequence is that the inference based on the “best” model can be misleading—either overestimating or underestimating, due to poor representative of the real distribution of the data. In addition, different methods, criteria, and even small changes in the data can result in very different final models, thus prediction accuracy of mode selection is reduced [23].

Model averaging has been widely recognized as a solution when model uncertainty is high and interferes with the quality of the prediction. By combining a set of competing models and wisely choosing the weights, model averaging can even out the overestimation and underestimation, therefore often leads to a more reliable result than the individual prediction. With model averaging, no assumption that a true model exists needs to be made, and inferences could be made about all candidate models. Hence, this method is preferred when uncertainty arises in the model selection process [24].

It has been shown that averaging models tend to yield better predictions than single models in the classical setting where the sample size is at least one order of magnitude greater than the number of predictors [16], how well it performs in high-dimensional regression, in which the number of predictors exceeds the sample size, is still under investigation. Recently, [2] proposed a two-step model averaging with cross-validation (MCV) procedure. The method consists of two steps: the first step is to construct candidate models by marginal correlation; the second step is to find optimal model weights by delete-one cross-validation. The main feature of MCV lies on the fact that the standard constraint of the model weights summing up to 1 is relaxed to the model weights can be vary freely between 0 and 1, and it is claimed that this relaxation lowers the prediction error.

The MCV approach is one of the only few references and is the first study that removes the weight restriction, thus marks a significant step toward the development in the high-dimensional frequentist model averaging. Nonetheless, we have several concerns with this method. First, the model construction step of MCV employs the marginal correlation to measure the strength of association between predictors and the response. Hence, it can miss some significant variables that are associated with the response conditionally but not marginally. Furthermore, the marginal correlation can be misleading when there exist non-trivial correlations among the predictors [20]. As a result, week candidate models could be involved and be given fairly large weights while strong candidate models could have small weights, and predictive inference is impaired. Second, we find the arguments in the paper regarding why the restriction of total weights summing to 1 should be removed are not convincing. For example, Ando and Li remarked *“consider the extreme case that the predictors are uncorrelated with each other and the noise variance is ignorable. The predictors from each model become uncorrelated with each other as well and the optimal combined predictor is the sum of all model predictors, implying that the optimal weight assignment should be (1, 1, ..., ). Thus, the total weight should be equal to M, not 1.”* This statement is problematic as for this special case, each model should be assigned the weight of $\frac{1}{M}$ if there are *M* of them, thus the total weight is still 1. Considering such point, more scrutiny is needed on whether relaxing the total weight constraint will lower the prediction error. Third, numerical study was conducted in that paper to demonstrate the favorable performance of MCV, but the same original data is employed for both model building and model validation, and the mean squared error (MSE) is calculated as the performance measure. A result of this model development process is that the MSE will tend to understate the inherent variability in making future predictions from the selected model [17], therefore the predictive capability of MCV needs to be re-calibrated by the collection of new data.

To address the above concerns of MCV and to further enhance the predictive ability, we develop a two-stage model averaging procedure for high-dimensional linear regression. In the first step, a high-dimensional variable selection method such as LASSO is employed to screen redundant predictors and to construct candidate models. In the second step, the jackknife cross-validation with the conventional constraint of total weights summing to 1 is applied to optimize model weights for averaging. The proposed method makes full use of the strengths of both model selection and averaging, meanwhile it is computational feasible since each candidate model and its corresponding weight are determined in the low-dimensional setting and the quadratic programming is utilized in the cross-validation. We conduct simulations and a real data example, the results illustrate that the proposed approach is efficient in forecasting and outperforms the existing predicting methodologies.

The remainder of the article is organized as follows. “Method” section describes the problem setting and an improved two-stage high-dimensional model averaging procedure is developed. We present a simulation study in “Simulations” section and provide a real data example in “Riboflavin Data Analysis” section for empirically examining the effectiveness of the proposed method. The paper ends with discussion and future research directions.

## Method

Given the dataset of *n* observations, a linear regression model takes the form of

2.1 $$\begin{aligned} y_i=\beta _1 x_{i1}+\cdots +\beta _p x_{ip}+\epsilon _i, \qquad i=1, 2,\ldots , n, \end{aligned}$$

where $y_i$ is the response in the *i*th trial, $x_{i1},\ldots , x_{ip}$ are the predictors, $\beta _1,\ldots , \beta _p$ are the regression coefficients, and $\epsilon _i$ is the error term. Here, we assume: 1) the independent error $\epsilon _i$ has mean zero and finite variance $\sigma ^2$; 2) the number of predictors *p* exceeds the sample size *n*; 3) only a subset of predictors have contributions in predicting the response.

Alternatively, in matrix form, model (2.1) can be written as

$$\begin{aligned} \mathbf{y }=\mathbf{X }{\varvec{\beta }}+{\varvec{\epsilon }}, \end{aligned}$$

where

$$\begin{aligned} \mathbf{y }& = {} \left( \begin{array}{c} {y_1}\\ \cdot \\ \cdot \\ \cdot \\ {y_n}\\ \end{array}\right) ,\quad {\varvec{\beta }}= \left( \begin{array}{c} {\beta _1}\\ \cdot \\ \cdot \\ {\beta _p}\\ \end{array} \right) , \qquad {\varvec{\epsilon }}= \left( \begin{array}{c} {\epsilon _1}\\ \cdot \\ \cdot \\ \cdot \\ \cdot \\ {\epsilon _n} \end{array} \right) ,\\ \mathbf{X }& = {} \left( \begin{array}{c} {\mathbf{X }_1}\\ \cdot \\ \cdot \\ {\mathbf{X }_n} \end{array}\right) = \begin{pmatrix} x_{11} &{} x_{12} &{} \ldots &{} x_{1p} \\ x_{21} &{} x_{22} &{} \ldots &{} x_{2p} \\ \vdots &{} \vdots &{} &{} \vdots \\ x_{n1} &{} x_{n2} &{} \ldots &{} x_{np} \end{pmatrix}. \end{aligned}$$

For model (2.1), we will develop a two-stage model averaging method to improve the predictive performance for high-dimensional regression.

### Model construction step

For the high-dimensional linear models, [2] proposed a two-stage model averaging procedure named MCV. The procedure first divides *p* predictors into $K + 1$ groups by the absolute marginal correlations between all predictors and the response. Let model $M_k$ consist of the predictors with marginal correlations falling into the *k*th group. The first group has the highest values, and the $(K + 1)$th group has values closest to 0 and is then discarded. Thus the number of candidate models is *K*. Each model can also be written in matrix form $\mathbf{y }=\mathbf{X }_k{\varvec{\beta }}_k+{\varvec{\epsilon }}$, for $k=1,\ldots , K$. Given candidate models whose number of predictors is smaller than the sample size, the regression coefficients are estimated by the least-squares method ${\hat{\varvec{\beta }}}_k=(\mathbf{X }'_k\mathbf{X }_k)^{-1}\mathbf{X }'_k\mathbf{y }$ and the predicted value ${\hat{\varvec{y}}}_k=\mathbf{X }_k{\hat{\varvec{\beta }}}_k$.

As discussed in "Introduction" section, the marginal correlation could provide misleading results in sorting variables and preparing candidate models therefore reduces prediction accuracy. To prepare proper candidate models ready for averaging, in the first step of the proposed method we consider some representative measurements which can simultaneously rank and select variables.

The first competitor is Distance Correlation. It is a measure of dependence between random vectors introduced by [22]. For all distributions with finite first moments, such method provides a robust way measuring and testing dependence by correlation of distances. The distance correlation test is implemented by the *energy* package in R. To find candidate models, we partition all predictors into different groups by the p-values of the test. The first group has the lowest values, and the last group has largest values and then is discarded.

Two well-known variable selection approaches are also considered for comparison. Ridge Regression [15] is a way to create a parsimonious model when the number of predictors exceeds the number of observations, or when multicollinearity exists in predictors. The predictors are sorted and chosen by the method proposed by Cule *et al.* [7] and can be conducted by the *ridge* package in R. Similar to Ridge Regression, LASSO [23] also works in a similar fashion the only difference is of the penalty term. LASSO is performed by *ncvreg* package in R, the importance of predictors is based on the coefficients magnitude, and those whose coefficients shrunk to 0 are abandoned.

Random Forest [4] is also under consideration. It is an ensemble learning method for classification, regression and other tasks that operate by constructing a multitude of decision trees. Random Forest can be used to rank the importance of variables and to conduct variable selection in regression. The technique was described in the papers by Genuer, Poggi and Tuleau-Malot [10, 11], and is implemented in the R package *VSURF*.

To evaluate the prediction performance, we adopt a simulation setting from Ando and Li [2]. The detailed structure of the simulation study is given below.

*Simulation 1*. Set the sample size $n=100, 200$ and the number of predictors $p=500, 1000, 2000$. Set the number of significant predictors $s=20, 50, 100$, and generate the coefficients $\beta _j$ from Unif(-1, 1). The predictors $x_{ij}, i=1, \ldots , n, j=1, \ldots , p$ are generated independently from Unif(-1, 1) as well. Finally, the error terms $\epsilon _i$ are generated from N$(0, \sigma =0.1, 1, 2)$. Therefore, in total there are $2*3*3*3=54$ combinations of $(n, p, s, \sigma )$.

To compare the methods in a fair manner, after candidate models are constructed in step 1, in step 2 the same delete-one cross-validation from MCV is used to optimize the model weights. In each run, we simulate a training set with *n* observations from the linear regression model in (2.1) for each combination. All the model building and averaging are done on the training set. We also collect an independent test data set of $n^*=1000$ observations to compute the mean of the squared prediction error (MSPE), MSPE=$\frac{\sum _{i=1}^{n^*} (y_i-{\hat{y}}_i)^2}{n^*}$. We repeat this process 100 times, and report the means and standard deviations of MSPE for each method.

**Table 1:** Simulated results for simulation 1, comparing the means and standard deviations (in the parentheses) of MSPE

Setting	MACOR	DISCOR	RIDGE	LASSO	FOREST
n100 p500 s20 σ.1	.905 (.3834)	.943 (.387)	.838 (.355)	**.0153 (.003)**	.965 (.385)
n100 p500 s20 σ1	2.603 (.469)	2.648 (.490)	2.629 (.469)	**2.462 (.381)**	2.715 (.442)
n100 p500 s20 σ 2	6.785 (.525)	**6.742 (.648)**	6.904 (.541)	7.094 (.886)	6.987 (.536)
n100 p500 s50 σ.1	4.458 (.760)	4.444 (.789)	**4.264 (.843)**	4.442 (1.03)	4.496 (.738)
n100 p500 s50 σ1	5.648 (.968)	5.715 (1.051)	**5.591 (.932)**	6.001 (1.083)	5.874 (.933)
n100 p500 s50 σ 2	**10.042 (1.092)**	10.103 (1.009)	10.221 (1.005)	10.771 (1.474)	10.050 (.945)
n100 p500 s100 σ.1	10.498 (1.292)	10.647 (1.397)	10.474 (1.402)	11.662 (1.768)	**10.442 (1.282)**
n100 p500 s100 σ1	11.498 (1.144)	11.879 (1.262)	**11.469 (1.092)**	12.885 (1.789)	11.701 (1.089)
n100 p500 s100 σ 2	15.652 (1.559)	15.639 (1.440)	**15.627 (1.490)**	17.047 (2.338)	15.622 (1.404)
n100 p1000 s20 σ.1	1.221 (.524)	1.189 (.489)	1.259 (.528)	**.064 (.346)**	1.270 (.481)
n100 p1000 s20 σ1	2.945 (.433)	**2.845 (.459)**	2.979 (.424)	2.867 (.496)	3.062 (.473)
n100 p1000 s20 σ2	**6.855 (.613)**	6.889 (.616)	6.868 (.571)	7.135 (.780)	6.996 (.635)
n100 p1000 s50 σ.1	4.874 (.779)	4.874 (.820)	**4.869 (.786)**	5.117 (1.114)	5.049 (.831)
n100 p1000 s50 σ1	6.192 (1.007)	**6.107 (1.005)**	6.219 (.963)	6.705 (1.359)	6.348 (1.006)
n100 p1000 s50 σ2	10.173 (.995)	10.179 (1.028)	**10.112 (.873)**	10.716 (1.433)	10.384 (.859)
n100 p1000 s100 σ.1	**11.163 (1.463)**	11.246 (1.439)	11.182 (1.339)	12.759 (2.017)	11.421 (1.531)
n100 p1000 s100 σ1	**12.380 (1.304)**	12.538 (1.143)	12.409 (1.165)	13.809 (1.841)	12.763 (1.393)
n100 p1000 s100 σ2	**16.815 (1.343)**	17.038 (1.574)	16.943 (1.332)	18.969 (2.574)	17.231 (1.563)
n100 p2000 s20 σ.1	1.758 (.569)	1.634 (.565)	1.782 (.574)	**.258 (.722)**	1.801 (.565)
n100 p2000 s20 σ1	3.041 (.446)	2.936 (.512)	3.088 (.529)	**2.874 (.583)**	3.081 (.469)
n100 p2000 s20 σ2	7.068 (.554)	7.166 (.592)	**7.058 (.578)**	7.651 (.985)	7.305 (.586)
n100 p2000 s50 σ.1	5.439 (.895)	**5.326 (.942)**	5.439 (.845)	5.699 (1.029)	5.599 (.922)
n100 p2000 s50 σ1	6.765 (.968)	**6.705 (1.004)**	6.808 (.936)	7.304 (1.180)	7.072 (.993)
n100 p2000 s50 σ2	**10.364 (.951)**	10.382 (.989)	10.469 (1.029)	11.316 (1.196)	10.639 (1.051)
n100 p2000 s100 σ.1	11.672 (1.241)	**11.588 (1.282)**	11.665 (1.329)	13.039 (1.588)	12.212 (1.294)
n100p2000s100σ1	**12.878 (1.205)**	13.089 (1.311)	13.104 (1.209)	14.229 (1.816)	13.523 (1.363)
n100 p2000 s100 σ2	**16.717 (1.233)**	16.799 (1.275)	16.867 (1.317)	18.999 (1.967)	17.074 (1.483)
n200 p500 s20 σ.1	.229 (.132)	.315 (.186)	.178 (.110)	**.012 (.001)**	.253 (.148)
n200 p500 s20 σ1	1.787 (.298)	1.794 (.312)	1.784 (.272)	**1.724 (.179)**	1.838 (.284)
n200 p500 s20 σ2	5.878 (.535)	**5.869 (.543)**	6.014 (.549)	6.078 (.659)	5.986 (.503)
n200 p500 s50 σ.1	2.098 (.654)	2.397 (.642)	1.663 (.685)	**.020 (.006)**	2.242 (.649)
n200 p500 s50 σ1	4.017 (.619)	4.246 (.658)	3.743 (.731)	**2.899 (.585)**	4.112 (.653)
n200 p500 s50 σ2	8.364 (.754)	8.511 (.738)	**8.321 (.738)**	8.641 (1.206)	8.455 (.801)
n200 p500 s100 σ.1	7.973 (1.338)	8.213 (1.254)	6.986 (1.493)	**6.836 (1.608)**	7.941 (1.381)
n200 p500 s100 σ1	9.123 (1.019)	9.439 (1.096)	8.723 (1.162)	**8.559 (1.266)**	9.232 (1.045)
n200 p500 s100 σ2	13.662 (1.452)	13.801 (1.352)	**13.350 (1.411)**	14.299 (1.699)	13.651 (1.359)
n200 p1000 s20 σ.1	.299 (.187)	.331 (.180)	.253 (.171)	**.0120 (.001)**	.332 (.197)
n200 p1000 s20 σ1	2.021 (.283)	1.991 (.279)	2.024 (.272)	**1.809 (.195)**	2.056 (.260)
n200 p1000 s20 σ2	6.416 (.554)	**6.357 (.528)**	6.536 (.552)	6.499 (.952)	6.463 (.576)
n200p1000s50 σ.1	3.046 (.849)	3.261 (.851)	2.848 (.897)	**.023 (.008)**	3.347 (.866)
n200 p1000 s50 σ1	4.771 (.694)	4.771 (.726)	4.676 (.735)	**3.869 (.882)**	4.969 (.729)
n200 p1000 s50 σ2	**9.089 (.918)**	9.093 (.954)	9.159 (.912)	9.323 (1.034)	9.152 (.920)
n200 p1000 s100 σ.1	8.786 (.975)	8.831 (.899)	**8.555 (.989)**	8.627 (1.375)	9.147 (.981)
n200 p1000 s100 σ1	10.224 (1.005)	10.290 (.981)	**10.036 (.940)**	10.628 (1.442)	10.509 (1.121)
n200 p1000 s100 σ2	14.143 (1.287)	14.219 (1.386)	**14.095 (1.313)**	14.976 (1.674)	14.474 (1.371)
n200 p2000 s20 σ.1	.419 (.249)	.432 (.279)	.429 (.276)	**.012 (.002)**	.473 (.266)
n200 p2000 s20 σ1	2.413 (.416)	2.288 (.368)	2.465 (.407)	**1.907 (.228)**	2.404 (.399)
n200 p2000 s20 σ2	6.593 (.470)	**6.556 (.519)**	6.653 (.454)	6.684 (.723)	6.641 (.533)
n200 p2000 s50 σ.1	3.729 (.874)	3.607 (.751)	3.731 (.825)	**.419 (1.050)**	3.900 (.711)
n200 p2000 s50 σ1	5.354 (.852)	5.089 (.877)	5.330 (.862)	**4.518 (1.012)**	5.465 (.949)
n200 p2000 s50 σ2	9.707 (.763)	**9.583 (.805)**	9.827 (.763)	10.054 (1.255)	9.972 (.798)
n200 p2000 s100 σ.1	9.693 (1.046)	**9.584 (1.137)**	9.686 (.980)	10.119 (1.456)	10.275 (1.169)
n200 p2000 s100 σ1	10.863 (1.194)	10.983 (1.052)	**10.578 (1.161)**	11.092 (1.609)	11.163 (1.292)
n200 p2000 s100 σ2	15.006 (1.164)	15.057 (1.145)	**14.948 (1.126)**	15.852 (1.129)	15.462 (1.169)

Bold values indicate the smallest MSPE in each scenario

MARCOR, DISCOR, RIDGE, LASSO, FOREST denote the method of Marginal Correlation, Distance Correlation, Ridge Regression, LASSO, Random Forest, respectively

Table 1 summarizes the simulation results. Several observations can be made from this table. First, the predictive performance is affected by several factors, including the sample size, number of predictors, number of significant predictors, and the distribution of the error terms. More specifically, the prediction accuracy increases as *n* increases, *p*, *s*, or $\sigma$ decreases. Second, out of the 54 scenarios, marginal correlation, Distance Correlation, Ridge Regression, LASSO, Random Forest perform the best 9/11/14/19/1 times, respectively. It seems that each method has its unique merits, none of the 5 methods can universally dominate the other competitors. The overall performance of LASSO appears to be the best, Ridge Regression is a close second. To explore the methods in more detail, we find that LASSO often performs favorably when *n* is large, or when *s* and $\sigma$ are small, yet ridge regression tends to be more stable among different cases. In summary, the simulation results show that using a shrinkage variable selection method to construct candidate models is preferable to the marginal correlation in the model construction step for model averaging.

### Model weighting step

After the candidate models and their corresponding least-squares predicted values $\{{\hat{\varvec{y}}}_1,\ldots , {\hat{\varvec{y}}}_K \}$ are obtained, the second stage of model averaging is to determine the model weights. For the MCV method, the weight vector ${\varvec{w}}$ is optimized by minimizing the delete-one cross-validation criterion

2.2 $$\begin{aligned} {\hat{\varvec{w}}}= \text {CV}({\varvec{w}})= ({\varvec{y}}-{\tilde{\varvec{y}}})'({\varvec{y}}-{\tilde{\varvec{y}}}), \end{aligned}$$

where ${\tilde{\varvec{y}}}=\sum _{k=1}^{K} w_k{\tilde{\varvec{y}}}_k$, ${\tilde{\varvec{y}}}_k=({\tilde{y}}^{(1)}_k, \ldots , {\tilde{y}}^{(n)}_k)$ be an *n*-dimensional vector, and ${\tilde{y}}^{(i)}_k$, is the predicted value ${\hat{y}}^{(i)}_k$ computed without the *i*th observation. Here, the restriction of $\sum _{k=1}^{K} w_k=1$ is removed, and the minimization of ${\hat{\varvec{w}}}$ can be easily solved by a quadratic programming. Following the similar idea, [3] further extended model averaging to high-dimensional generalized linear models.

To compare with the relaxed cross-validation criterion in MCV, we consider three weight choice alternates in which the conventional constraint of total weights summing to 1 is applied.

AIC [1] is a consistent estimator of the Kullback-Leibler discrepancy between the distribution that generated the data and the model that approximates it. Suppose there are *K* models, AIC$_k=-2$log$(L_k)+2p_k$, where $L_k$ is the maximized likelihood function and $p_k$ is the number of parameters under the *k*-th model. [5] advocated to use the MAIC for determining the weights

$$\begin{aligned} w_k=\frac{\mathrm{exp}(-AIC_k/2)}{\sum _{k=1}^{K}\mathrm{exp}(-AIC_k/2)}, \qquad k=1, \ldots , K. \end{aligned}$$

More recently, [12] proposed a least-squares model average estimator with model weights selected by minimizing Mallows’ $C_p$ criterion [21]. The Mallows’ criterion for the model averaging estimator (MMA) is

$$\begin{aligned} C_n({\varvec{w}})= \left( {\varvec{y}}-\mathbf{X }\varvec{\hat{\beta }}\right) '\left( {\varvec{y}}-\mathbf{X }\varvec{\hat{\beta }}\right) +2\sigma ^2k({\varvec{w}}), \end{aligned}$$

where $k({\varvec{w}})$ is the effective number of parameters, and $\sigma ^2$ can be estimated by a sample variance from a “large” approximating model. Hansen showed that the model average estimator that minimizes the Mallows criterion also minimizes the squared error in large samples.

In addition, [13] suggested to select the weights by minimizing a deleted-one cross-validation criterion, and the method is termed as the jackknife model averaging (JMA). The same method is adopted in MCV as expressed in (2.2). However, JMA requires $\sum _{k=1}^{K} w_k=1$ while such conventional restriction is relaxed in MCV. This criterion is quadratic in the weights, so computation is a plausible application of quadratic programming. It is also shown that the JMA estimator is asymptotically optimal in achieving the lowest possible expected squared error.

To compare all the weight determining methods, we conduct another simulation study.

*Simulation 2*. We use the same setting and data generating process in *Simulation 1*. The same candidate models are constructed from the training data, all the methods described in this session are used to determine the model weights. An independent test data set of $n^*=1000$ observations is generated to compute the prediction accuracy measured by the MSPE. We repeat the iteration 100 times.

**Table 2:** Simulated results for simulation 2, comparing the means and standard deviations (in the parentheses) of MSPE

Setting	CV	MAIC	MMA	JMA
n100 p500 s20 σ.1	.856 (.413)	**.829 (.423)**	.841 (.418)	.854 (.418)
n100 p500 s20 σ1	2.575 (.403)	2.649 (.485)	2.474 (.378)	**2.471 (.379)**
n100 p500 s20 σ 2	6.877 (.699)	6.839 (.888)	6.183 (.711)	**6.177 (.708)**
n100 p500 s50 σ.1	**4.337 (.881)**	5.080 (1.003)	4.493 (.835)	4.492 (.840)
n100 p500 s50 σ1	5.922 (.918)	6.822 (1.1122)	5.919 (.907)	**5.916 (.907)**
n100 p500 s50 σ 2	9.919 (1.029)	10.593 (1.286)	9.374 (1.128)	**9.328 (1.029)**
n100 p500 s100 σ.1	10.581 (1.227)	11.924 (1.461)	**10.376 (1.075)**	10.377 (1.073)
n100p500s100σ1	11.696 (1.354)	12.829 (1.530)	11.205 (1.248)	**11.196 (1.254)**
n100 p500 s100 σ2	15.640 (1.197)	16.939 (1.633)	14.693 (1.183)	**14.682 (1.160)**
n100 p1000 s20 σ.1	1.329 (.467)	1.286 (.585)	**1.264 (.497)**	1.267 (.494)
n100 p1000 s20 σ1	2.946 (.396)	2.990 (.553)	2.739 (.431)	**2.732 (.417)**
n100 p1000 s20 σ2	7.084 (.494)	7.065 (.664)	6.276 (.537)	**6.260 (.509)**
n100 p1000 s50 σ.1	4.947 (.774)	5.541 (.977)	4.895 (.743)	**4.895 (.744)**
n100 p1000 s50 σ1	6.145 (.634)	6.633 (.799)	5.870 (.617)	**5.869 (.616)**
n100 p1000 s50 σ2	10.429 (.870)	10.805 (1.222)	9.419 (.771)	**9.414 (.768)**
n100 p1000 s100 σ.1	11.385 (1.481)	12.309 (1.595)	**10.857 (1.294)**	10.861 (1.291)
n100 p1000 s100 σ1	12.515 (1.621)	13.700 (1.928)	**11.931 (1.624)**	11.932 (1.629)
n100 p1000 s100 σ2	16.333 (1.826)	17.324 (2.306)	15.147 (1.609)	**15.143 (1.611)**
n100 p2000 s20 σ.1	1.728 (.575)	1.575 (.627)	**1.519 (.560)**	1.519 (.559)
n100 p2000 s20 σ1	3.115 (.502)	3.113 (.681)	**2.823 (.506)**	2.833 (.506)
n100 p2000 s20 σ2	7.283 (.651)	7.559 (.746)	**6.490 (.573)**	6.491 (.575)
n100 p2000 s50 σ.1	5.345 (.895)	5.815 (1.209)	5.092 (.885)	**5.092 (.886)**
n100 p2000 s50 σ1	6.880 (.803)	7.57 (1.207)	6.523 (.805)	**6.521 (.806)**
n100 p2000 s50 σ2	10.925 (1.118)	11.403 (1.383)	**9.954 (.998)**	9.955 (.998)
n100 p2000 s100 σ.1	11.894 (1.136)	13.166 (1.814)	11.280 (1.159)	**11.278 (1.162)**
n100p2000s100σ1	13.014 (1.047)	14.281 (1.667)	**12.243 (1.028)**	12.245 (1.028)
n100 p2000 s100 σ2	16.564v1.558)	17.597 (2.241)	15.282 (1.459)	**15.282 (1.460)**
n200 p500 s20 σ.1	.254 (.151)	**.245 (.143)**	.247 (.146)	.251 (.149)
n200 p500 s20 σ1	1.724 (.251)	**1.679 (.260)**	1.686 (.247)	1.693 (.249)
n200 p500 s20 σ2	5.859 (.428)	5.823 (.499)	5.512 (.424)	**5.499 (.415)**
n200 p500 s50 σ.1	2.226 (.683)	2.224 (.726)	**2.221 (.709)**	2.234 (.706)
n200 p500 s50 σ1	**3.933 (.683)**	4.061 (.811)	3.964 (.710)	3.959 (.718)
n200 p500 s50 σ2	8.453 (.761)	8.988 (.979)	8.203 (.799)	**8.170 (.757)**
n200 p500 s100 σ.1	**8.055 (1.101)**	9.544 (1.538)	8.567 (1.251)	8.538 (1.267)
n200p500 s100 σ1	**9.269 (.832)**	10.965 (1.212)	9.758 (.874)	9.722 (.859)
n200 p500 s100 σ2	13.247 (1.304)	14.661 (1.640)	13.144 (1.247)	**13.076 (1.249)**
n200 p1000 s20 σ.1	.330 (.217)	**.306 (.198)**	.318 (.211)	.325 (.214)
n200 p1000 s20 σ1	2.008 (.309)	**1.802 (.302)**	1.841 (.289)	1.841 (.286)
n200 p1000 s20 σ2	6.319 (.531)	6.191 (.633)	5.714 (.480)	**5.713 (.479)**
n200p1000 s50 σ.1	**2.757 (.841)**	2.844 (1.047)	2.775 (.919)	2.803 (.905)
n200 p1000 s50 σ1	**4.699 (.729)**	5.080 (.893)	4.716 (.747)	4.717 (.745)
n200 p1000 s50 σ2	9.028 (.824)	9.438 (1.062)	8.515 (.752)	**8.514 (.743)**
n200p1000s100σ.1	**9.070 (1.087)**	10.523 (1.293)	9.366 (1.105)	9.366 (1.106)
n200 p1000 s100 σ1	**9.881 (1.102)**	11.395 (1.368)	10.034 (1.046)	10.028 (1.049)
n200 p1000 s100 σ2	14.765 (1.169)	16.065 (1.569)	**14.097 (1.159)**	14.100 (1.161)
n200 p2000 s20 σ.1	.409 (.267)	**.334 (.208)**	.369 (.226)	.373 (.225)
n200 p2000 s20 σ1	2.454 (.362)	**2.113 (.416)**	2.151 (.357)	2.152 (.356)
n200 p2000 s20 σ2	6.529 (.536)	6.355 (.622)	5.791 (.484)	**5.789 (.481)**
n200 p2000 s50 σ.1	3.736 (.939)	3.760 (1.066)	**3.644 (.961)**	3.653 (.948)
n200 p2000 s50 σ1	5.443 (.714)	5.693 (.915)	5.227 (.710)	**5.227 (.710)**
n200 p2000 s50 σ2	9.577 (.759)	10.098 (1.009)	**8.923 (.722)**	8.925 (.723)
n200 p2000 s100 σ.1	**9.813 (1.085)**	11.147 (1.312)	9.842 (1.064)	9.842 (1.064)
n200 p2000 s100 σ1	11.309 (1.278)	12.557 (1.691)	11.091 (1.254)	**11.091 (1.255)**
n200 p2000 s100 σ2	15.286 (1.056)	16.467 (1.499)	**14.388 (1.076)**	14.390 (1.079)

Bold values indicate the smallest MSPE in each scenario

Table 2 compares the means and standard deviations of MSPE for each method. Out of the 54 scenarios, the relaxed cross-validation (refers to CV in the table), MAIC, MMA, JMA perform the best 9/7/14/24 times. The overall performance of JMA appears to be the best, MMA performs similarly but slightly worse. More importantly, we find that in most cases (45 out of 54), relaxing the total weight constraint does not lower the prediction error. The results confirm with our concern in "Introduction" section.

### The improved two-stage high-dimensional model averaging

Based on the results from the numerical experiments, we propose an improved model average (IMA) procedure, which contains the following two steps:

*Stage 1*: Construct candidate models by a high-dimensional penalized variable selection method (LASSO or its variants, such as SCAD [8] and ALASSO [26]).

- Run the penalized regression for the high-dimensional data and obtain the LASSO estimate for each predictor.
- Partition all *p* predictors into $K+1$ groups by the magnitude of the estimates. The first group has the largest absolute values of LASSO estimates, and the $(K + 1)$th group contains the predictors whose coefficients are already shrunk to 0. We drop the $(K + 1)$th group, thus the number of candidate models is *K*.
- For each model $M_k$, $k=1,\ldots , K$, $\mathbf{y }=\mathbf{X }_k{\varvec{\beta }}_k+{\varvec{\epsilon }}$, and the least-squares prediction is ${\hat{\varvec{y}}}_k=\mathbf{X }_k{\hat{\varvec{\beta }}}_k$.

*Stage 2*: Optimize the model weights by the standard jackknife cross-validation criterion by Hansen and Racine [13].

- Let ${\tilde{\varvec{y}}}_k=({\tilde{y}}^{(1)}_k, \ldots , {\tilde{y}}^{(n)}_k)$ be an *n*-dimensional vector, where ${\tilde{y}}^{(i)}_k$, is the predicted value ${\hat{y}}^{(i)}_k$ computed without the *i*th observation.
- Let ${\varvec{w}}=(w_1, \ldots , w_k)$ be the weight vector for the *K* models, then the jackknife predictor is
  $$\begin{aligned} {\tilde{\varvec{y}}}=\sum _{k=1}^{K} w_k {\tilde{y}}_k. \end{aligned}$$
- The optimal estimate of ${\varvec{w}}$ is obtained by minimizing the cross-validation
  $$\begin{aligned} {\hat{\varvec{w}}}= \text {CV}({\varvec{w}})= ({\varvec{y}}-{\tilde{\varvec{y}}})'({\varvec{y}}-{\tilde{\varvec{y}}}), \end{aligned}$$
  where the standard restriction $\sum _{k=1}^{K} w_k=1$ is applied.
- Finally, the model averaging predicted value ${\hat{\varvec{y}}}$ is expressed as
  $$\begin{aligned} {\hat{\varvec{y}}}=\sum _{k=1}^{K} {\hat{w}}_k {\hat{\varvec{y}}}_k . \end{aligned}$$

#### *Remark*

As a referee points out, ranking is an essential component of the out-performance. By ranking all the predictors based on the magnitude of the estimates, the models proposed in Stage 1 are not equally competitive. The first few models are likely to be more informative than the last few models because of the ordering of regressors. Therefore, assigning larger weights to the first few models and smaller weights to the last few models tend to improve the predictive performance. Note that [2] also ranked the predictors in the MCV method by using the marginal correlation. Moreover, we have conducted some simulation experiments, the results show that (although not shown in the article) ranking predictors into *K* different groups has lower prediction errors than a random assignment of non-zero predictors.

#### *Remark*

The overall performance of the proposed method depends on the choice of parameter *K* which is the total number of models. Determining the optimal value of *K* is similar to finding the best tuning parameter $\lambda$ in penalized variable selection. For each candidate value of *K*, a group of models is constructed and the best model weights are determined. The optimal *K* is the one with the lowest cross-validation score. Note that the specification of *K* may vary case by case, here we propose a practical strategy for optimizing the choice of *K*. The number of predictors $p_k$ in each model is set to be the same, $p_k = n \times q$, where n is the sample size, q is a value between 0 and 1. First, we set *T* to be the number of predictors whose LASSO estimates are non-zero. Because the total number of predictors from *K* models is equal to $K \times p_k$, it is desirable for $K \times q \times n$ to be no greater than T. Subject to this restriction, we choose K and q whose cross-validation prediction error is the smallest.

## Simulations

To assess the performance of the improved model averaging (IMA) method, we compare our approach with some existing model selection and averaging methods on simulated data. These competitors include MAIC [5], LASSO [23], MCV [2], and SISSCAD (a combination of sure independence screening [9] and SCAD [8]).

In this simulation study, we follow the settings of Ando and Li [2], 6 model settings are considered.

- Set the sample size $n=50$ and the number of predictors $p=2000$. Let the number of true predictors $s=50$, the true predictors $x_i$ are spaced evenly, $i=40(j-1)+1, j=1, \ldots , 50.$ Further, the true coefficients $\beta _j$ are generated from N(0, 0.5), and the design matrix ${\varvec{X}}$ is generated from N$(0, \Sigma = \rho ^{|i-j|})$, where $\rho =0.6$. Finally, the error terms $\epsilon _i$ are generated from N$(0, \sigma =0.2)$.
- Under the same setting (a), *n* is increased to 100.
- Under the same setting (a), the value of $\rho$ is decreased to 0.
- Under the same setting (a), the standard deviation of the error term $\sigma$ follows Unif(2.1, 2.3).
- Under the same setting (d), *n* is increased to 100.
- Under the same setting (d), the value of $\rho$ is decreased to 0.

**Fig. 1:**
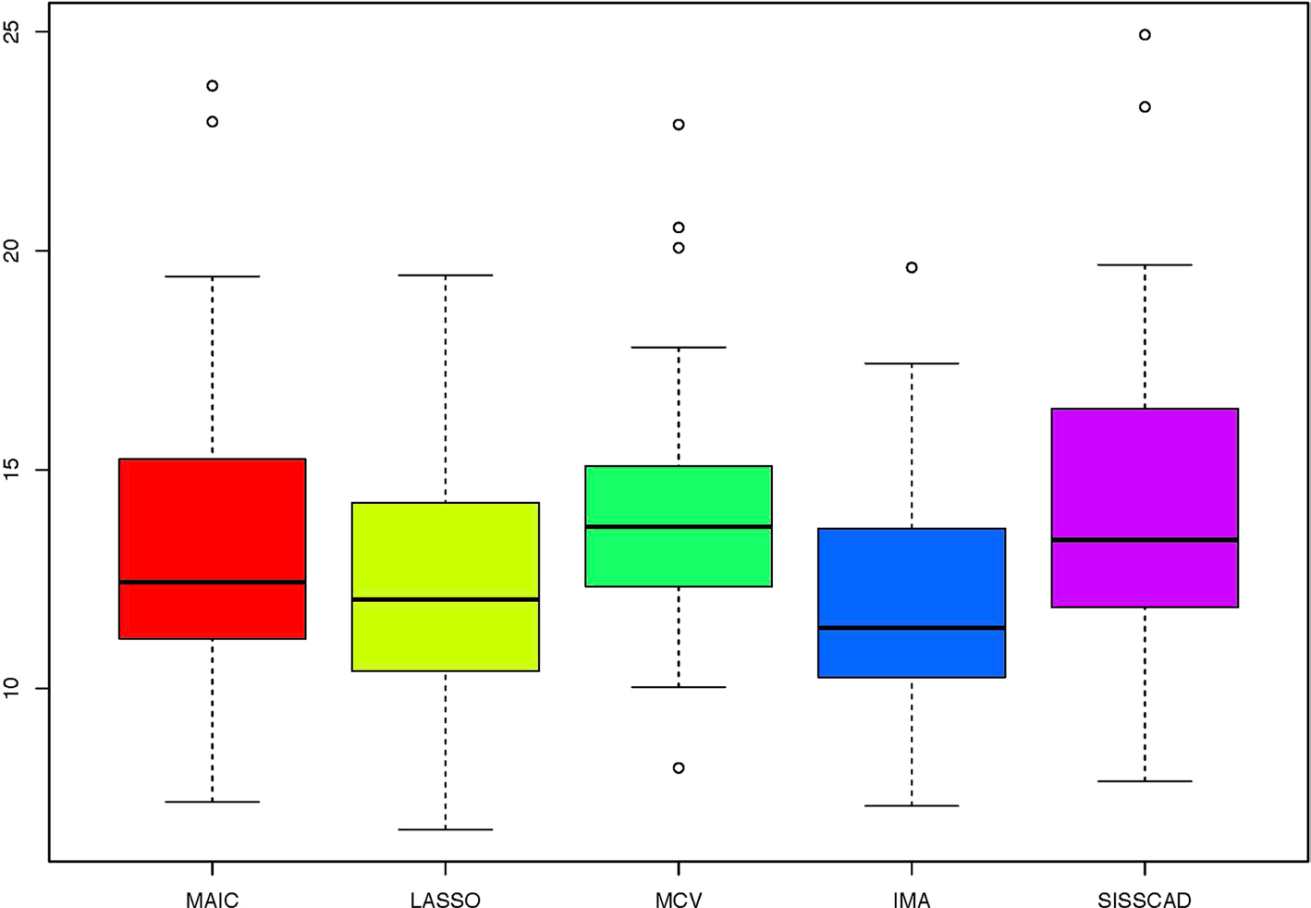
Boxplots of MSPEs for Setting (a)

**Fig. 2:**
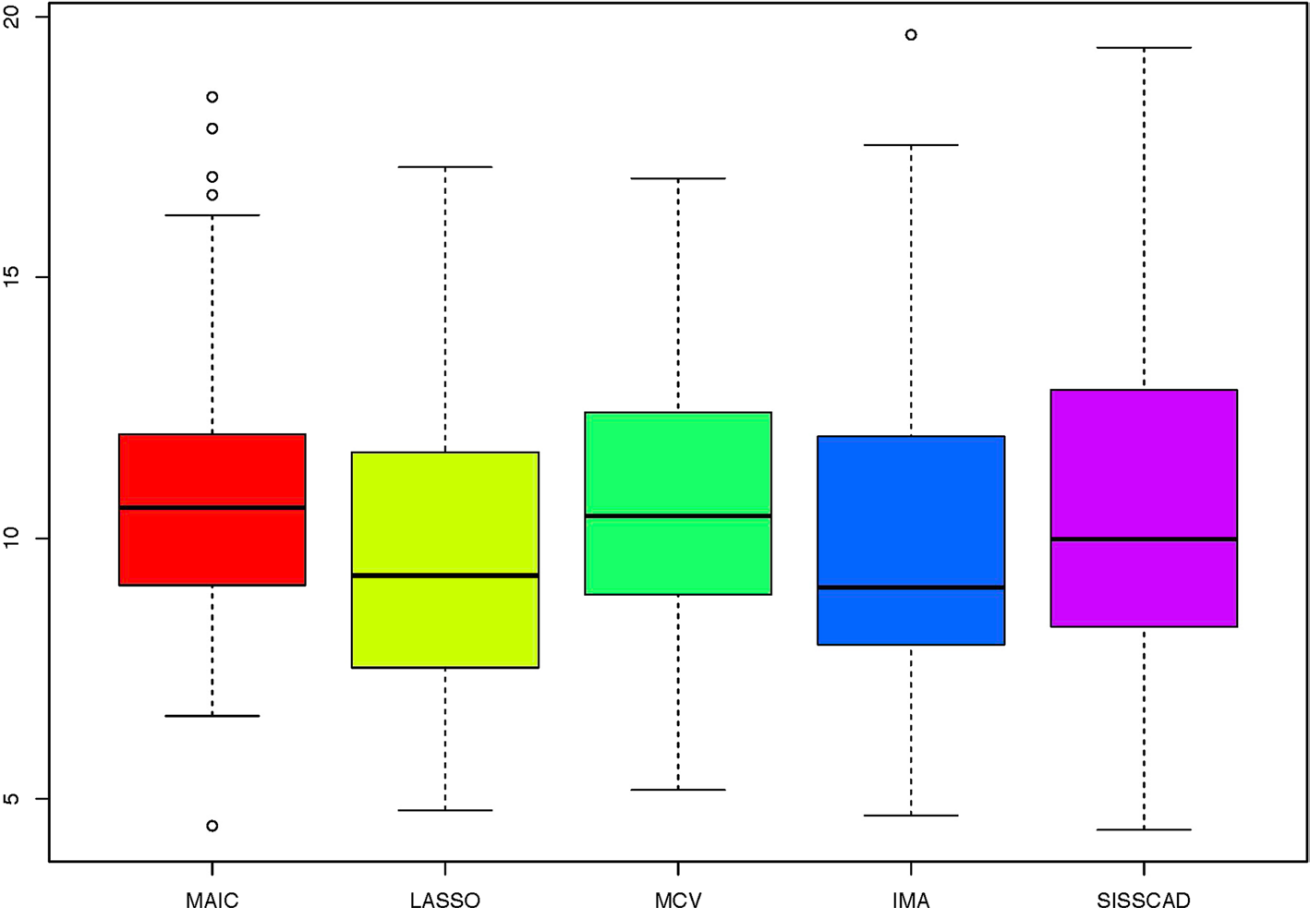
Boxplots of MSPEs for Setting (b)

**Fig. 3:**
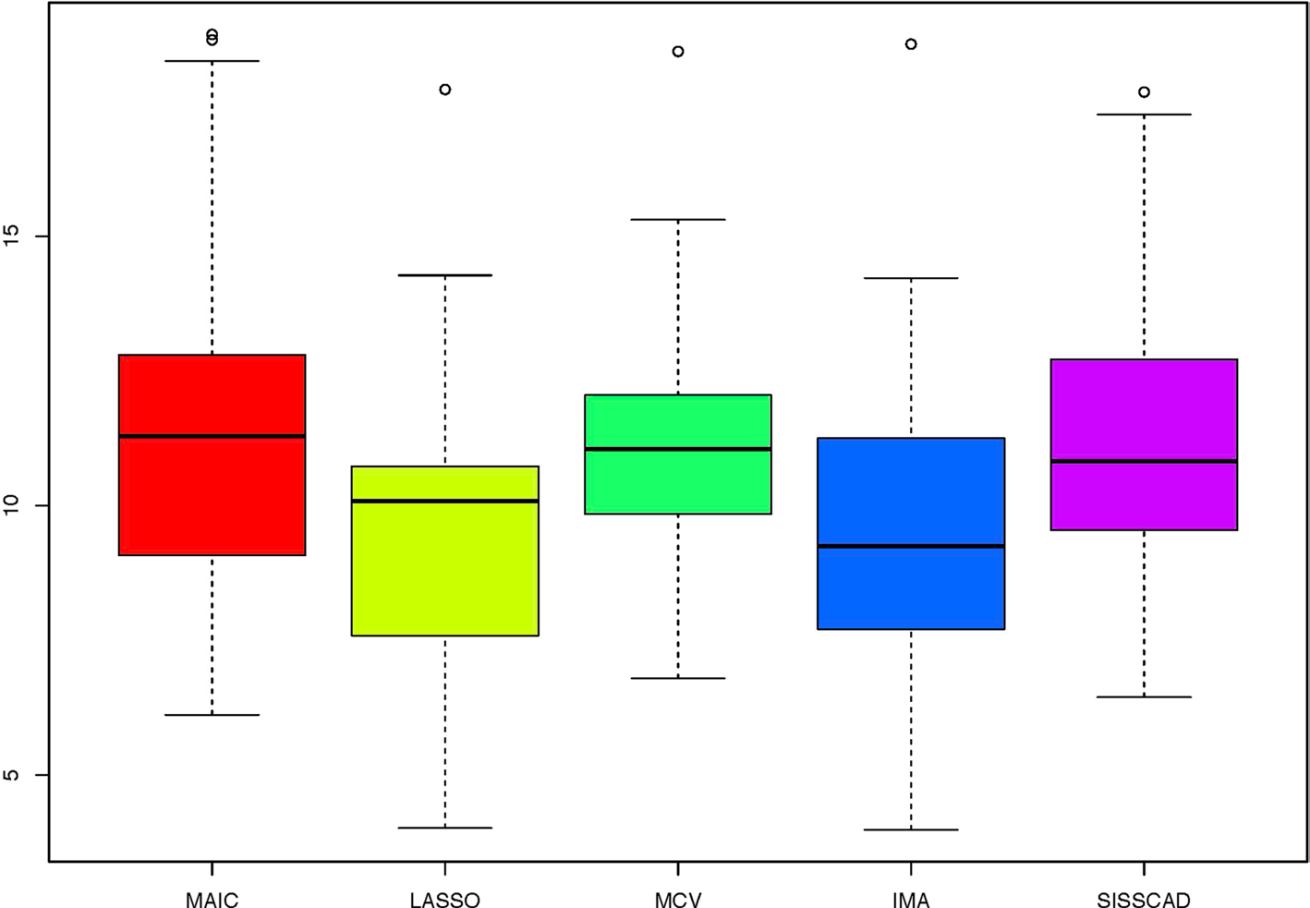
Boxplots of MSPEs for Setting (c)

**Fig. 4:**
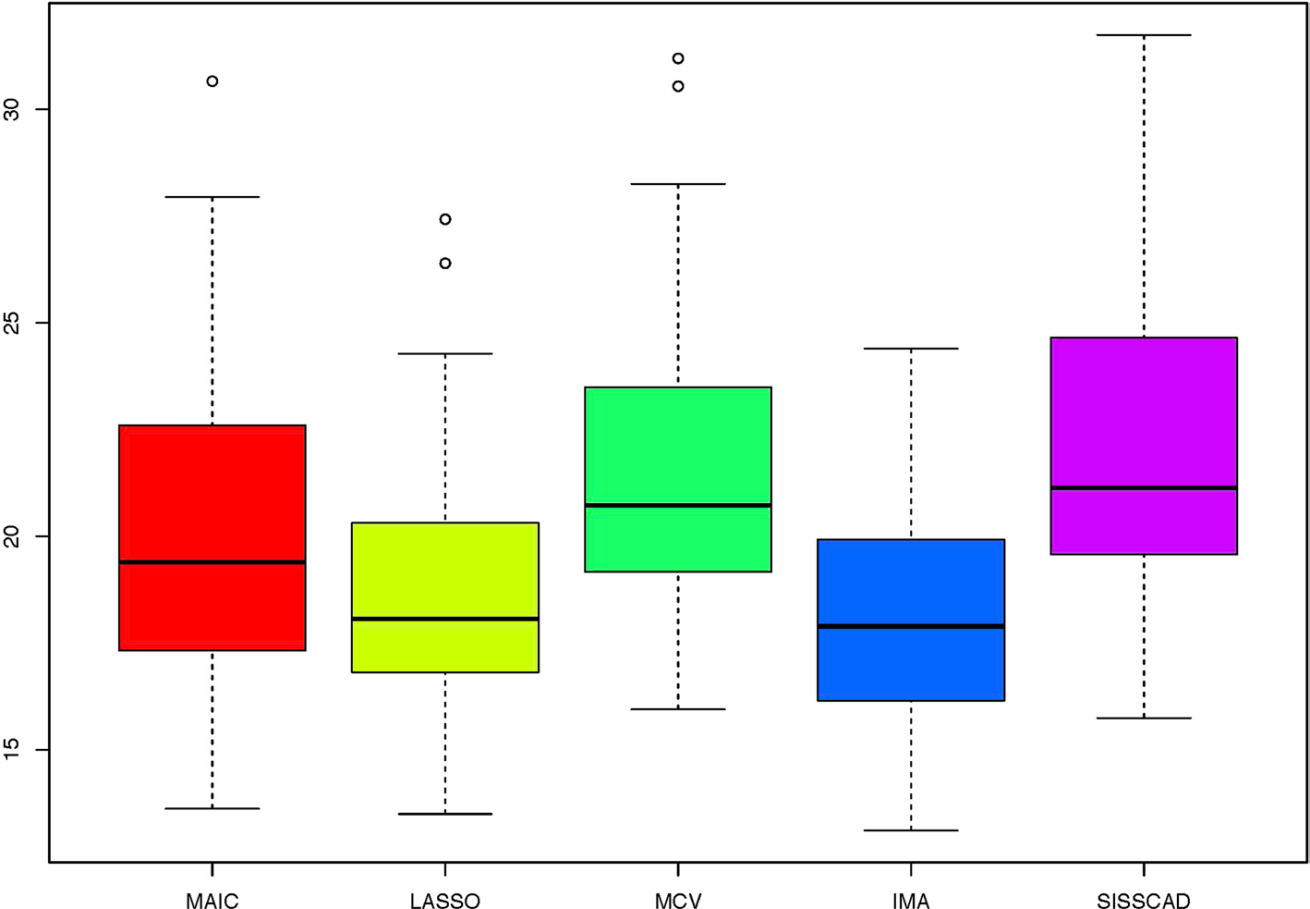
Boxplots of MSPEs for Setting (d)

**Fig. 5:**
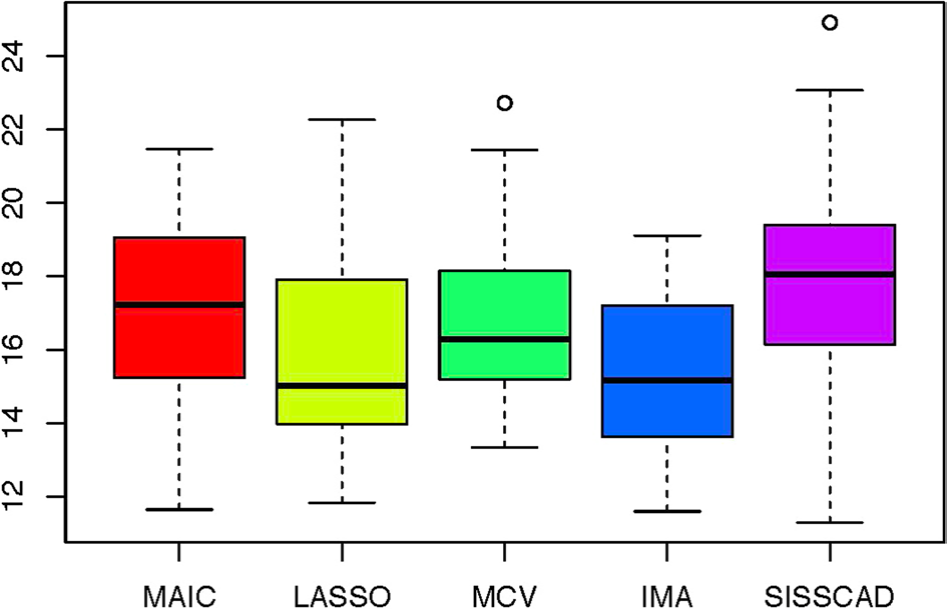
Boxplots of MSPEs for Setting (e)

**Fig. 6:**
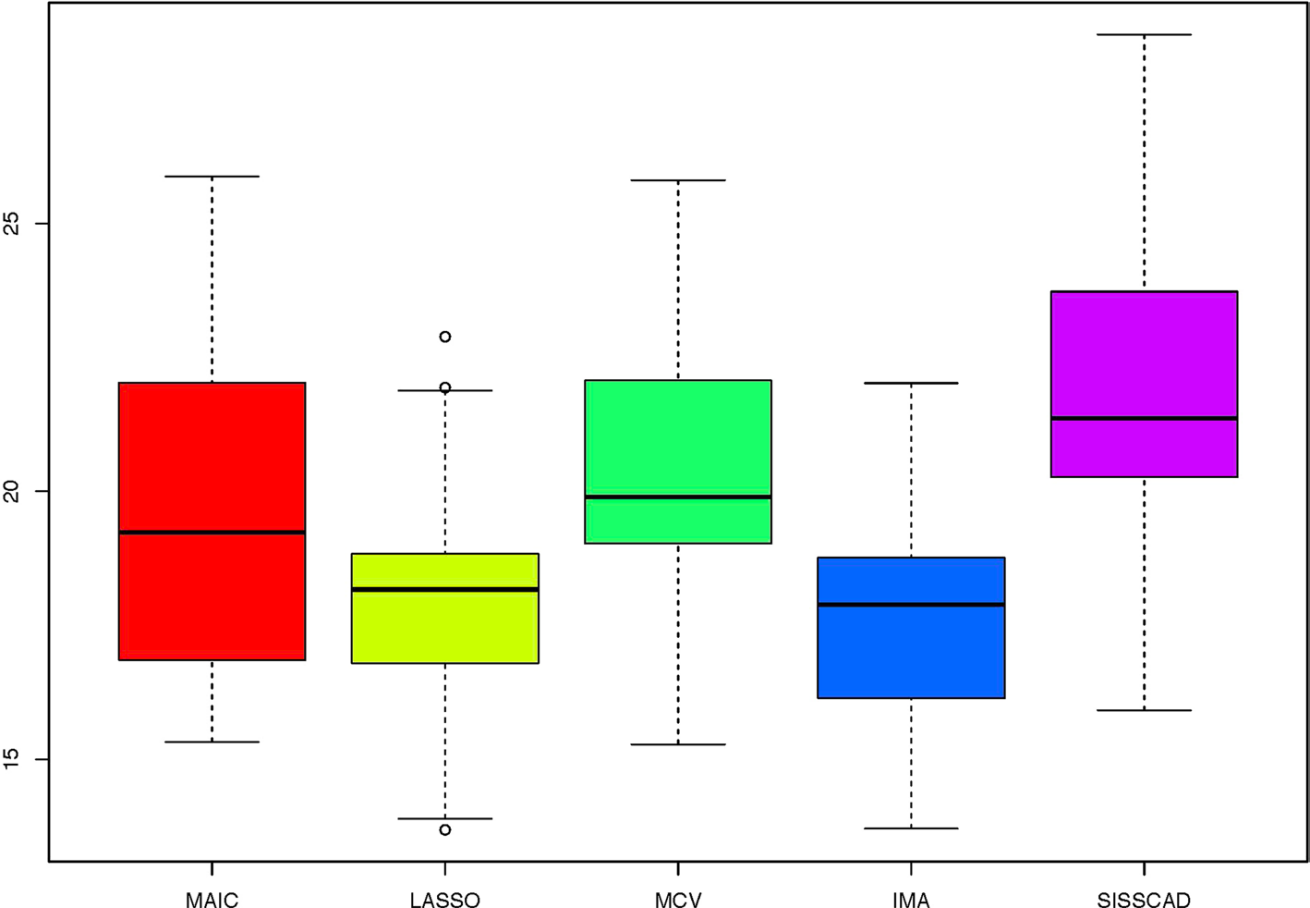
Boxplots of MSPEs for Setting (f)

For each setting, we generate $n^*=1000$ observations to compute MSPE. Figures 1, 2, 3, 4, 5 and 6 show the boxplots of MSPEs after 100 simulation runs under settings (a) to (f). As we can see, increasing the sample size ((a) V.S (b) and (d) V.S. (e)), and decreasing the correlation $\rho$ in the design matrix ${\varvec{X}}$ ((a) V.S (c) and (d) V.S. (f)), both tend to decrease the prediction error. In addition, the proposed procedure IMA yields the smallest median of MSPE in most settings, and achieves stable performance by having the short length of the boxplots. The results demonstrate that IMA performs favorably in comparison with other methods. In particular, the median of MSPE of IMA is at least 20% lower than that of MCV, we remark that the IMA method outperforms MCV, and thus significantly improves the prediction accuracy and stability of model averaging for high-dimensional linear regression.

**Table 3:** Averaged computational time (in seconds) and corresponding standard deviations (in the parentheses) for each method

Setting	MAIC	LASSO	MCV	IMA	SISSCAD
Setting (a)	13.773	.397	13.767	.457	.035
	(7.012)	(.020)	(7.010)	(.046)	(.002)
Setting (b)	2.237	.672	2.220	.749	.047
	(.612)	(.064)	(.598)	(.082)	(.006)
Setting (c)	9.757	.405	9.727	.472	.037
	(3.624)	(.023)	(3.612)	(.050)	(.002)
Setting (d)	11.600	.298	11.599	.510	.037
	(2.993)	(.069)	(2.992)	(.072)	(.003)
Setting (e)	2.065	.403	2.069	.922	.047
	(.418)	(.075)	(.429)	(.097)	(.001)
Setting (f)	9.667	.325	9.595	.594	.038
	(2.727)	(.069)	(2.706)	(.079)	(.005)

Table 3 compares the computational time required for each method under the settings (a) to (f). The computations are conducted using R version 3.5.0 on the Owens clusters at the Ohio Supercomputer Center (OSC). After 100 simulation runs, the averaged time (in seconds) and corresponding standard deviations are given. Several observations can be seen from this table. First, with the fixed number of predictors *p* and the correlation $\rho$, increasing the sample size *n* will decrease the computing time. Second, with the fixed number of predictors *p* and the sample size *n*, decreasing the correlation $\rho$ will decrease the computing time. Third, in general, model selection methods (LASSO, SISSCAD) have less computing time than the model averaging procedures (MAIC, MCV, IMA). Last, the proposed method IMA is much faster than other model averaging competitors and is comparable to model selection methods regarding the computational cost.

## Riboflavin data analysis

To further explore the practical behavior of the proposed method, we consider the riboflavin data with $n=71$ observations in Bühlmann and Mandozzi (2014 [6]). The response variable *y* is the logarithm of the riboflavin production rate, the predictors are the gene expression levels for 4088 genes. The methods to be included in the comparison experiment are: IMA, MAIC, SCAD, MCV, and RMSA (another recent model averaging procedure by Lin et al. [19]. We randomly divide the data into a training set with 50 observations and a test set with the remaining 21. In each time, *p* = 500 or 1000 genes are chosen randomly from the totality of all genes, the process is repeated 100 times.

The results for the Riboflavin data are presented in Table 4. Similar to the results in the simulation study, the prediction errors of IMA are substantially smaller than those of the other methods, and the computing time is pretty competitive. It is also noted that RMSA performs well in this real example, while the amount of computation required for such method is considerably high. Therefore, compared to RMSA, IMA not only has slightly better performance in prediction accuracy, but also enjoys much lower computing cost.

**Table 4:** Results for the Riboflavin data, comparing the means and standard deviations (in the parentheses below) of MSPE, and the averaged computational time (in seconds) and corresponding standard deviations (in the parentheses below) for each method

Case	MAIC	LASSO	SCAD	MCV	IMA	RMSA
$$p=500$$						
MSPE	.489	.475	.469	1.294	.371	.402
	(.166)	(.179)	(.163)	(1.612)	(.127)	(.155)
Running Time	12.379	.111	.130	12.371	.172	.761
	(10.698)	(.008)	(.017)	(10.709)	(.054)	(.080)
$$p=1000$$						
MSPE	.384	.375	.372	1.713	.316	.322
	(.123)	(.189)	(.173)	(2.993)	(.130)	(.127)
Running Time	12.079	.176	.210	12.113	.248	.795
	(16.953)	(.032)	(.057)	(17.007)	(.068)	(.062)

## Discussion

To achieve a stable and improved prediction, we propose a novel two-stage model averaging procedure in high-dimensional linear regression models. The method uses variable selection to group predictors for model averaging and applies the standard jackknife cross-validation for optimizing weights.

Compared with the recent model averaging procedure (MCV, [2]), the proposed approach has better predictive performance, meanwhile it retains computational flexibility even for extra high-dimensional data. We conduct numerical studies including simulations and a real riboflavin data analysis, the results demonstrate that the proposed technique is quite efficient in forecasting and outperforms the existing methodologies in general.

Even though advantages of model averaging over model selection have been demonstrated in the low dimensional regression analysis, it is still unclear to a large extent when model averaging should be preferred is high-dimensional linear models. [25] proposed an index, PIE, to measure model selection instability in estimation. They suggested that: If PIE is bigger than 0.5, model averaging should be considered; if PIE is less than 0.4, model selection is likely to work better than the model combining methods. However, PIE does not function properly for high-dimensional data based on our investigation. In the future, we intend to develop a measure which can be used to guide practitioners for deciding which way to go: averaging or selection, when dealing with high-dimensional problems.

## Supplementary Information

**Additional file 1.** Riboflavin dataset.

## Data Availability

The riboflavin data analyzed during the current study is provided in a supplementary file.
